# Deficiency of the Fanconi anemia core complex protein FAAP100 results in severe Fanconi anemia

**DOI:** 10.1172/JCI185126

**Published:** 2025-04-17

**Authors:** Benjamin A. Harrison, Emma Mizrahi-Powell, John Pappas, Kristen Thomas, Subrahmanya Vasishta, Shripad Hebbar, Anju Shukla, Shalini S. Nayak, Tina K. Truong, Amy Woroch, Yara Kharbutli, Bruce D. Gelb, Cassie S. Mintz, Gilad D. Evrony, Agata Smogorzewska

**Affiliations:** 1 Laboratory of Genome Maintenance, The Rockefeller University, New York, New York, USA.; 2 Center for Human Genetics and Genomics, New York University Grossman School of Medicine, New York, New York, USA.; 3 Department of Pediatrics, Clinical Genetic Services, NYU Langone Medical Center, New York, New York, USA.; 4 Department of Pathology, NYU Langone Health, New York University School of Medicine, New York, New York, USA.; 5 Department of Medical Genetics and; 6 Department of Obstetrics and Gynecology, Kasturba Medical College, Manipal, Manipal Academy of Higher Education, Manipal, Karnataka, India.; 7 Department of Genetics and Genomic Sciences and; 8 Mindich Child Health and Development Institute and Department of Pediatrics, Icahn School of Medicine at Mount Sinai, New York, New York, USA.; 9 Department of Pediatrics, Department of Neuroscience and Physiology, Institute for Systems Genetics, Perlmutter Cancer Center, and Neuroscience Institute, New York University Grossman School of Medicine, New York, New York, USA.

**Keywords:** Development, Genetics, Bone marrow, DNA repair, Genetic diseases

## Abstract

Fanconi anemia (FA) is a rare genetic disease characterized by loss-of-function variants in any of the 22 previously identified genes (*FANCA*–*FANCW*) that encode proteins participating in the repair of DNA interstrand crosslinks (ICLs). Patient phenotypes are variable but may include developmental abnormalities, early-onset pancytopenia, and a predisposition to hematologic and solid tumors. Here, we describe 2 unrelated families with multiple pregnancy losses and offspring presenting with severe developmental and hematologic abnormalities leading to death in utero or in early life. Homozygous loss-of-function variants in *FAAP100* were identified in affected children of both families. The FAAP100 protein associates with FANCB and FANCL, the E3 ubiquitin ligase responsible for the monoubiquitination of FANCD2 and FANCI, which is necessary for FA pathway function. Patient-derived cells exhibited phenotypes consistent with FA. Expression of the WT *FAAP100* cDNA, but not the patient-derived variants, rescued the observed cellular phenotypes. This establishes FAAP100 deficiency as a cause of FA, with FAAP100 gaining an alias as FANCX. The extensive developmental malformations of individuals with *FAAP100* loss-of-function variants are among the most severe across previously described FA phenotypes, indicating that the FA pathway is essential for human development.

## Abstract 2

The authors identify *FAAP100* gene variants as causing a severe subtype of Fanconi anemia characterized by multi-organ developmental abnormalities and bone marrow deficiency at birth.

## Graphical abstract

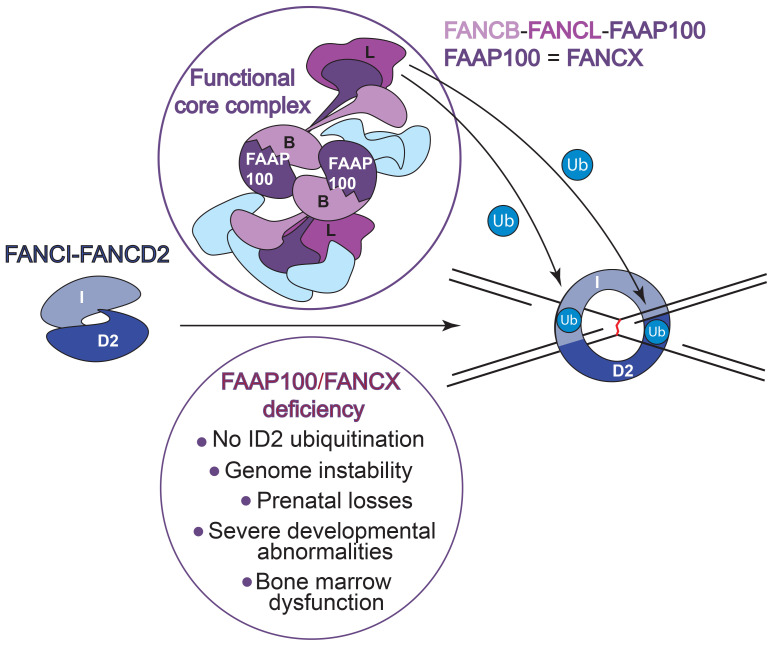

## Introduction

Fanconi anemia (FA) is a rare genetic disorder characterized by developmental defects, early-onset pancytopenia secondary to bone marrow failure, and a predisposition to leukemia and solid tumors (1–3). FA is caused by loss of any of at least 22 genes (*FANCA–FANCW*) that encode proteins in the FA repair pathway. FA is a recessive disease caused by biallelic loss-of-function variants, except for *FANCB* and *FANCR*, which are X-linked and de novo autosomal dominant, respectively (4–7).

The FA pathway repairs DNA interstrand crosslinks (ICLs) that covalently link the Watson and Crick strands of DNA, which pose a challenge for both DNA replication and transcription. ICLs can be caused by endogenous biological metabolites such as aldehydes as well as by exogenous crosslinking agents such as diepoxybutane (DEB), cisplatin (CDDP), or mitomycin C (MMC) (8–11). FA-deficient cells display spontaneous chromosomal breakage that is enhanced after treatment with DNA interstrand crosslinking agents, a finding that also serves as the basis for clinical diagnosis of FA (12).

FA-dependent ICL repair occurs during S-phase in a series of conserved steps that allow for faithful replication of the genome. The necessary step in ICL repair by the FA pathway is the monoubiquitination of the FANCI-FANCD2 heterodimer (hereafter referred to as the ID2 complex), which forms a clamp fully encircling the DNA at the site of an ICL (13–18). Monoubiquitination of ID2 is achieved by an E3 ubiquitin ligase, FANCL, which is part of an 8-protein core complex that is activated upon ICL sensing. Downstream steps in ICL lesion repair include nucleolytic unhooking of the ICL dependent on FANCP/SLX4 and ERCC4/FANCQ/XPF, translesion synthesis by translesion polymerases such as Pol zeta, and homologous recombination, which result in repaired dsDNA (19–26).

FAAP100 was identified as a component of the FA core complex through biochemical studies showing that it interacts with proteins in the FA core complex and is necessary for FA pathway function (27). More recent structural studies have elucidated that the core complex is initiated by the formation of a dimer of heterotrimers composed of FANCB-FANCL-FAAP100 (hereafter called the B-L-100 complex) (13, 28). They, in turn, serve as a scaffold for the other core complex proteins to associate in a nonsymmetric fashion, with only 1 FANCL being active in ubiquitination (13). FANCB-FAAP100 heterodimerization by their C-termini is postulated to be the first step in core complex assembly, with other proteins in the core complex projecting out from the core (13). The B-L-100 complex has been shown to be both necessary and sufficient for the monoubiquitination of FANCD2 in vitro (29).

The FA repair pathway is necessary in the majority of mammalian organs. Its deficiency has the potential to cause developmental abnormalities, early-onset bone marrow failure, infertility, and an increased risk for a variety of cancers (1, 30–32). However, FA is a highly heterogeneous disease with variable expressivity, and patients rarely manifest all possible clinical findings. The severity of a patient’s clinical presentation is typically correlated with the affected gene and the specific variant type. Patients with biallelic variants in breast cancer 2 (*BRCA2*, also known as *FANCD1*) and partner and localizer BRCA2 (*PALB2*, also known as *FANCN*) may present with characteristic FA developmental abnormalities and classically develop acute myeloid leukemias and embryonal tumors including medulloblastoma, neuroblastoma, and Wilms tumors. The severity of those presentations is thought to be due to the functions of the proteins coded by these 2 genes in general homology-directed repair (33–37). Among other FA genes, *FANCB* is associated with more severe phenotypes, including early-onset bone marrow failure and extensive developmental abnormalities, most notably vertebral anomalies, anal atresia, cardiac defects, tracheoesophageal fistula, esophageal atresia, renal and limb abnormalities, and hydrocephalus (VACTERL-H) (38–42). However, the clinical severity associated with *FANCB* is also linked to the variant type. The most severe phenotypes usually present in patients with whole-gene deletions of *FANCB*, whereas patients with point mutations in *FANCB* present with milder phenotypes such as later-onset hematological disease with no or fewer developmental abnormalities (43). Similarly, patients with hypomorphic variants in *FANCA*, the most commonly diagnosed FA disease–associated gene, might present later in life with cancer and infertility but no developmental abnormalities or bone marrow failure (44–48). Overall, the wide spectrum of severity among individuals with FA suggests that some FA-associated proteins (FAAPs) have not yet been associated with FA because their loss is incompatible with life, causing spontaneous abortion (SAB). Indeed, despite the central role of FAAP100 in the FA core complex, *FAAP100* has not yet been associated with human disease.

In this report, we describe 2 unrelated families found to have distinct loss-of-function variants in the *FAAP100* gene. The mothers of both families presented initially with numerous SABs, and the children who were carried to term in 1 family died soon after birth and in the second family died at 14 months of age. The neonates were found to have numerous physical anomalies, comprising many FA clinical findings described to date and also extending the clinical spectrum. Affected children in family 1 harbored a homozygous frameshift variant leading to loss of RNA and protein expression, while an affected child in family 2 had a homozygous stop-gain variant near the C-terminus, causing loss of only the 18 C-terminal amino acids. Through functional complementation, we show that the variants in *FAAP100* were causative for FA in both families. These results also highlight the importance of the C-terminus of FAAP100 for its function, likely in its ability to heterodimerize with FANCB to form the heart of the FA core complex. This study establishes FAAP100 as a new FA gene, now designated FANCX, whose deficiency leads to a severe FA phenotype comparable to that seen with complete deletion of *FANCB*.

## Results

### Identification of a homozygous variant in FAAP100 in a newborn with developmental abnormalities.

**Figure 1:**
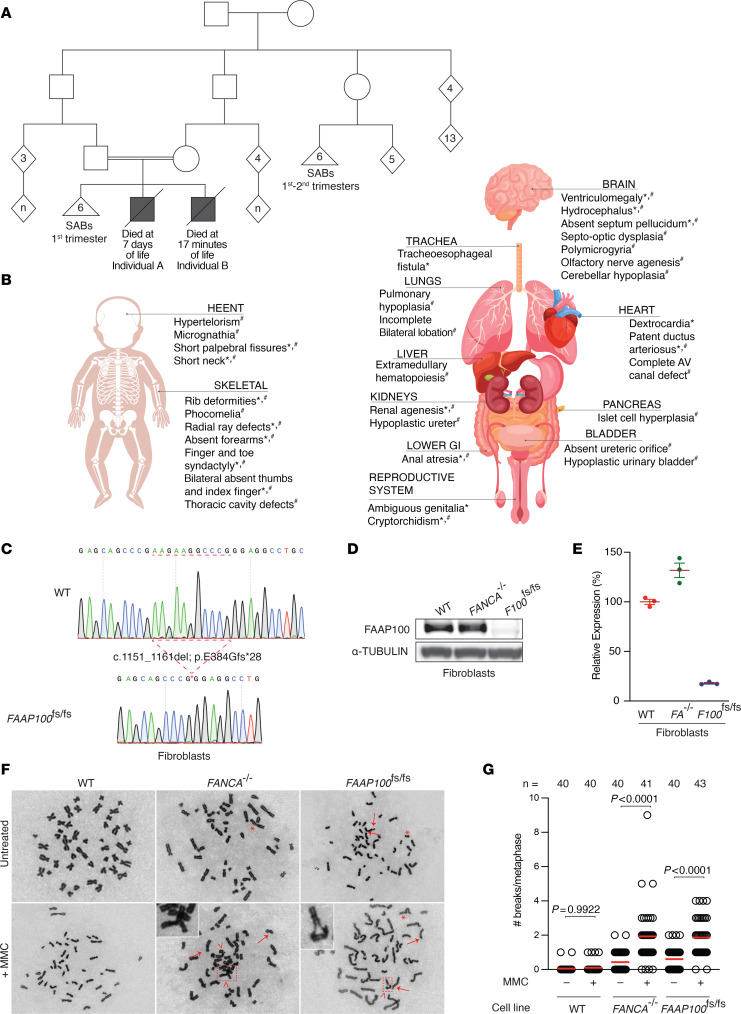
Characterization of organismal and cellular phenotypes in a family with *FAAP100* variants. (**A**) Pedigree of family 1 showing consanguinity with multiple SABs and neonatal deaths. (**B**) Summary of developmental abnormalities identified in individual A (*) and individual B (#). HEENT, head, eyes, ears, nose, and throat; GI, gastrointestinal. (**C**) Genotyping of exon 3 of *FAAP100* in individual B’s fibroblasts (RA3640) showing biallelic deletion of 11 nucleotides. (**D**) Representative immunoblot showing FAAP100 protein in WT (BJ) and *FANCA^–/–^* fibroblasts (RA3087) and no FAAP100 protein in individual B’s primary fibroblasts. (**E**) RT-qPCR of the *FAAP100* mRNA transcript in WT, *FANCA^–/–^* (*FA^–/–^*), and *F100*^fs/fs^ (individual B) fibroblasts showing a lack of transcript expression in individual B’s cell lines. (**F**) Representative images of metaphases. Arrows show breaks, asterisks show gaps, and arrowheads show chromosome radials. Metaphases were imaged using a ×63 oil immersion objective, and insets are a ×7.1 magnification of the selected chromosomes. (**G**) Quantification of spontaneous and MMC-induced chromosome breakage in individual B’s LCLs and control LCLs shown in **F**, with at least 40 metaphases per cell line. Experiments were conducted at least 3 times in biological replicates with consistent results for **D**, **E**, and **G**. Data from a representative experiment are shown and show the mean ± SEM. All *P* values were calculated using 1-way ANOVA.

A couple (family 1) who is consanguineous presented with a history of 8 pregnancies, 6 of which resulted in SAB and 2 that resulted in death of the infant soon after birth (Figure 1A). Both liveborn male children, designated individuals A and B, presented with severe congenital anomalies. Individual A was diagnosed by prenatal ultrasound with cerebral ventriculomegaly, absent right kidney, intrauterine growth restriction (IUGR), multiple limb abnormalities, and cardiac defects. A subsequent fetal echocardiogram identified hypoplasia of the proximal ascending aorta, a ventricular septal defect, and an aberrant subclavian artery. He was born at 39 weeks’ gestation and was promptly admitted to the neonatal intensive care unit. Physical examination was notable for birth weight and length below the first percentile, head circumference below the third percentile, bilateral short forearms, an absent thumb and second digit on the right hand, a thumb-like digit fused to the adjacent digit on the left hand with oligodactyly, toe syndactyly, microphthalmia, microtia, cleft palate, imperforate anus, microphallus with scrotal hypoplasia, and other congenital anomalies (Figure 1B and Supplemental Table 1; supplemental material available online with this article; https://doi.org/10.1172/JCI185126DS1). Postnatal echocardiograms showed dextrocardia, hypoplastic branch pulmonary arteries, a diminutive aortic valve, and a small mitral valve. Chest and extremity x-rays revealed several additional findings including absent bilateral radii, hypoplasia of the bilateral humeri, sacral hypoplasia with partial fusion of the sacral bodies, and various rib anomalies. Abdominal and pelvic ultrasound showed a solitary right kidney and undescended testes bilaterally without any evidence of Müllerian structures. Brain MRI revealed severe hydrocephalus, fused thalami, absent septum pellucidum, absent pituitary stalk, diffuse white matter volume loss, extremely thinned corpus callosum, and cystic dilation of the bilateral semicircular canals. Laboratory tests were notable for pancytopenia at birth that required several RBC and platelet transfusions and hypopituitarism (low cortisol, free T4, and free T3). Leukopenia, anemia, and thrombocytopenia persisted during the hospitalization. Further phenotypic details are provided in Figure 1B, Supplemental Tables 1 and 2, and Supplemental Text. On day 7 of life, the infant began to have declines in oxygen saturation and subsequent bradycardia, eventually leading to asystole and death. The parents declined an autopsy.

Initial genetic testing included a congenital heart disease (CHD) panel and rapid genome sequencing (rGS), neither of which identified any causative variants. Chromosome breakage studies were performed due to the phenotypic overlap with FA. The DEB-induced breakage of 2.94 breaks/chromosome was higher than an age-matched control of 0.06 breaks/chromosome, consistent with a diagnosis of FA. After individual A’s death, a custom genetic testing panel was ordered for his mother, targeting genes in the FA pathway. This panel identified a heterozygous frameshifting deletion variant of uncertain significance (VUS) in *FAAP100* (c.1151_1161del; p.E384Gfs*28; NM_025161.5; chr17:81,550,333-81,550,343del [hg38]; note, because of a repetitive sequence at this locus, the variant can also be annotated as chr17:81,550,338-81,550,348del [hg38]). Reanalysis of individual A’s rGS data revealed homozygosity for this variant. To date, the ClinVar database is devoid of known pathogenic variants in *FAAP100*.

Individual B’s prenatal ultrasound also revealed multiple anomalies, including severe IUGR, hydrocephalus, multicystic dysplastic kidneys, a contracted and hypoplastic urinary bladder, and an imperforate anus. Testing of chorionic villus samples revealed a normal karyotype, and the microarray was nondiagnostic. Chromosomal breakage studies failed because of poor tissue culture growth. Exome sequencing on amniocytes revealed homozygosity for the same *FAAP100* VUS identified in individual A and confirmed heterozygosity in both parents. Given the nondiagnostic clinical evaluation, the family was enrolled in the Undiagnosed Diseases Program (UDP) of the New York University Grossman School of Medicine.

Individual B was born at 40 weeks gestation with low tone, no spontaneous respiratory effort, and a heart rate of approximately 40 bpm. His heart rate declined further despite ventilation, and he died 17 minutes after birth. The infant’s birth weight and length were both below the first percentile, and his head circumference was below the third percentile. An autopsy revealed severe congenital anomalies, including phocomelia, absent thumbs and index fingers, thoracic cavity deformities, craniofacial anomalies, genitourinary abnormalities, an imperforate anus, severe pulmonary hypoplasia, and a complete atrioventricular (AV)canal defect. The brain was immature, with polymicrogyria, olfactory nerve agenesis, partial agenesis of the septum pellucidum, cerebellar hypoplasia, and ventriculomegaly. Notably, microscopic examination of rib bone marrow revealed erythroid hyperplasia (Supplemental Figure 1, A and B) and extramedullary hematopoiesis in the spleen and liver (Supplemental Figure 1C). See Figure 1B, Supplemental Table 1, and Supplemental Text for further phenotypic details.

Skin fibroblasts (cell line ID RA3640) and a lymphoblastoid cell line (LCL) (cell line ID RA3641) were derived from individual B at the time of autopsy. Both cell lines were found to harbor the same homozygous frameshift variant in *FAAP100* identified by clinical sequencing (Figure 1C and Supplemental Figure 1D) and are referred to hereafter as *FAAP100*^fs/fs^ cell lines. Immunoblotting for FAAP100 in *FAAP100*^fs/fs^ primary fibroblasts revealed a lack of protein expression, which was present in a positive control of WT foreskin fibroblasts (BJ cells) and *FANCA*-deficient patient-derived fibroblasts (RA3087) (Figure 1D). Reverse transcription quantitative PCR (RT-qPCR) in *FAAP100*^fs/fs^ fibroblasts and LCLs confirmed a lack of *FAAP100* mRNA expression in the patient’s cell lines. (Figure 1E and Supplemental Figure 1E).

### Patient-derived cells exhibit a FA phenotype.

**Figure 2:**
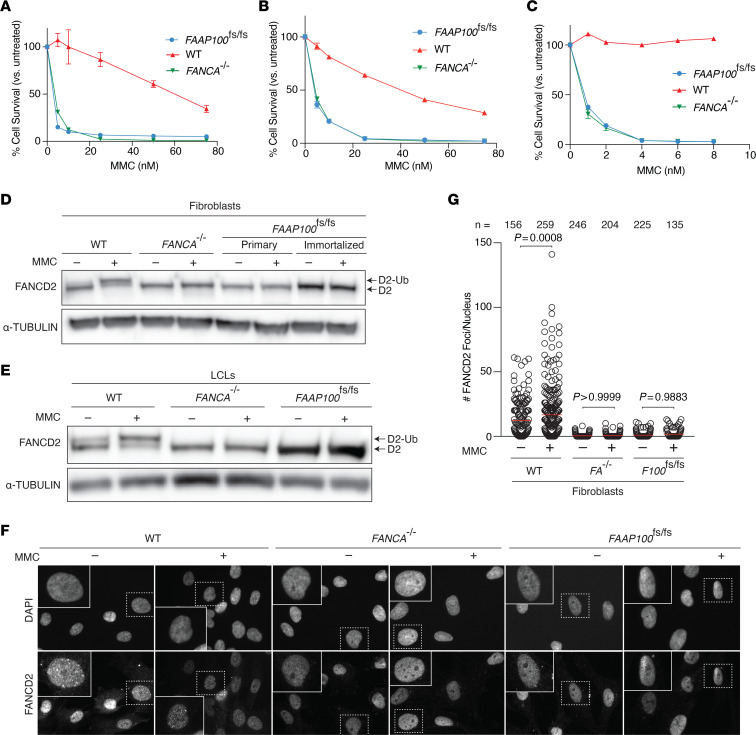
Further characterization of the cellular phenotype of FAAP100 deficiency. (**A**–**C**) Sensitivity of *FANCA^–/–^* and *FAAP100*^fs/fs^ cells to increasing doses of MMC in primary fibroblasts (**A**), E6E7 and hTERT immortalized fibroblasts (**B**), and EBV-immortalized LCLs (**C**), compared with control cells. (**D** and **E**) Representative immunoblot showing FANCD2 in the indicated (**D**) fibroblasts and (**E**) LCLs. D2-Ub indicated monoubiquitinated FANCD2. (**F**) Representative images of FANCD2 IF showing foci formation in WT but not *FANCA^–/–^* or *FAAP100*^fs/fs^ cells. Cells were imaged using a ×63 oil immersion objective, and inserts are a ×4.4 magnification of the selected nuclei shown in the insets. (**G**) Quantification of nuclear foci shown in **F** with at least 130 nuclei per cell line. Experiments were conducted at least 3 times in biological replicates with consistent results for **A**–**C** and **G**. Data from a representative experiment are shown. Data indicate the mean ± SEM. All *P* values were calculated using 1-way ANOVA.

As clinical chromosome breakage analysis was unsuccessful in individual B due to poor cell growth, the *FAAP100*^fs/fs^ fibroblasts and LCLs were assessed for both spontaneous and crosslinking agent MMC-induced breakage, which is characteristic of FA. Metaphase spreads of *FAAP100*^fs/fs^ cells had significant spontaneous and MMC-induced chromosome breaks, gaps, and radials at a level similar to that seen in *FANCA-*deficient cells (Figure 1, F and G). *FAAP100*^fs/fs^ primary fibroblasts, immortalized fibroblasts, and LCLs also showed hypersensitivity to MMC in growth assays, even at very low levels of DNA damage (Figure 2, A–C and Supplemental Figure 1F).

The *FAAP100*^fs/fs^ cells were also assessed for their ability to monoubiquitinate FANCD2 in response to MMC, another readout for FA repair pathway competency. Immunoblotting for FANCD2 in fibroblasts (Figure 2D) and LCLs (Figure 2E) with or without MMC treatment revealed that the *FAAP100*^fs/fs^ cells lacked FANCD2 ubiquitination in response to MMC, similar to *FANCA-*deficient fibroblasts. In parallel, nuclear FANCD2 foci formation was interrogated by immunofluorescence (IF) in *FAAP100*^fs/fs^ cells. As expected, neither *FAAP100*^fs/fs^ nor *FANCA*-deficient cells exhibited nuclear FANCD2 foci formation in response to MMC treatment, whereas WT fibroblasts retained this ability (Figure 2, F and G). These data demonstrate that individual B’s cells lacked FA repair pathway activity, consistent with a diagnosis of FA.

### Complementation with WT FAAP100 cDNA rescues observed cellular phenotypes of patient-derived cells.

**Figure 3:**
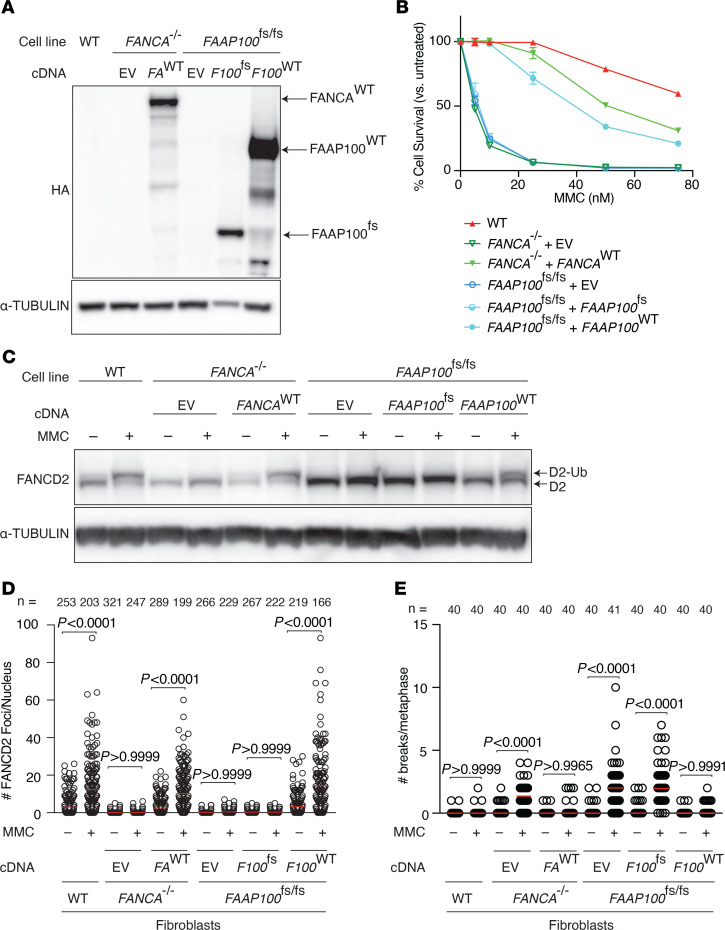
Expression of the WT *FAAP100* cDNA in *FANCA^–/–^* and *FAAP100*^fs/fs^ cells. (**A**) Immunoblot with HA-tagged antibodies in cells complemented with empty vector (EV), individual B–derived mutant cDNA (*FAAP100*^fs^), and WT cDNA (*FAAP100*^WT^). (**B**) Sensitivity of complemented cells to increasing doses of MMC was rescued only in cells expressing the WT *FAAP100* cDNA construct. (**C**) Immunoblot showing restoration of FANCD2 monoubiquitination in cells complemented with WT, but not individual B–derived mutant cDNA. (**D**) Quantification of FANCD2 nuclear foci formation by IF in the indicated cells with at least 160 nuclei per cell line. (**E**) Quantification of chromosome breaks in the indicated cells in at least 40 metaphases per cell line. Experiments were conducted at least 3 times in biological replicates with consistent results for **B**, **D**, and **E**. Data from a representative experiment are shown. Data indicate the mean ± SEM. All *P* values were calculated using 1-way ANOVA.

To determine whether *FAAP100* is the gene responsible for the cellular phenotype observed in patient-derived cells, *FAAP100*^fs/fs^ fibroblasts were complemented with WT N-terminally HA-FLAG tagged *FAAP100* cDNA, *FAAP100*^fs^ cDNA, or vector control and were assayed for rescue of the FA phenotype. Expression of the respective constructs was confirmed by immunoblotting for HA and FAAP100, which showed the expected protein size (Figure 3A and Supplemental Figure 2A). Overexpression of WT *FAAP100*, but not the frameshift mutant, rescued cellular hypersensitivity to MMC (Figure 3B), lack of FANCD2 ubiquitination (Figure 3C), lack of nuclear FANCD2 foci formation (Figure 3D), and chromosomal instability (Figure 3E and Supplemental Figure 2B). These results confirm that FAAP100 deficiency was the cause of FA in family 1.

Given the severity of the developmental phenotypes observed in family 1, we reanalyzed individual B’s exome sequencing data to search for variants that may intensify the FA phenotype. This reanalysis did not reveal any notable variants in *ALDH2*, *ADH5*, or *ALDH9A1*, which code for proteins that detoxify aldehydes that otherwise can form DNA ICLs (8, 49–52). Thus, the severe phenotypes seen in family 1 are unlikely to be due to variants in genes related to aldehyde detoxification.

### Variants in FAAP100 found in a second family with similarly severe FA.

A nonconsanguineous couple (family 2) presented with a history of 2 SABs. One female neonate (individual C) was carried to term and presented with congenital anomalies including bilateral microtia, reduced radius size in both forearms, absence of both thumbs, and a right radial club hand. At 5 months of age, the infant presented with a fever and at that time was diagnosed with tetralogy of Fallot, a single ectopic kidney, and microcornea. Failure to thrive was noted, and at 14 months of age, the child experienced a series of febrile seizures that resulted in death due to respiratory failure. Genetic testing of the child, including karyotype and FISH for 22q11.2 microdeletion syndrome, were nondiagnostic.

**Figure 4:**
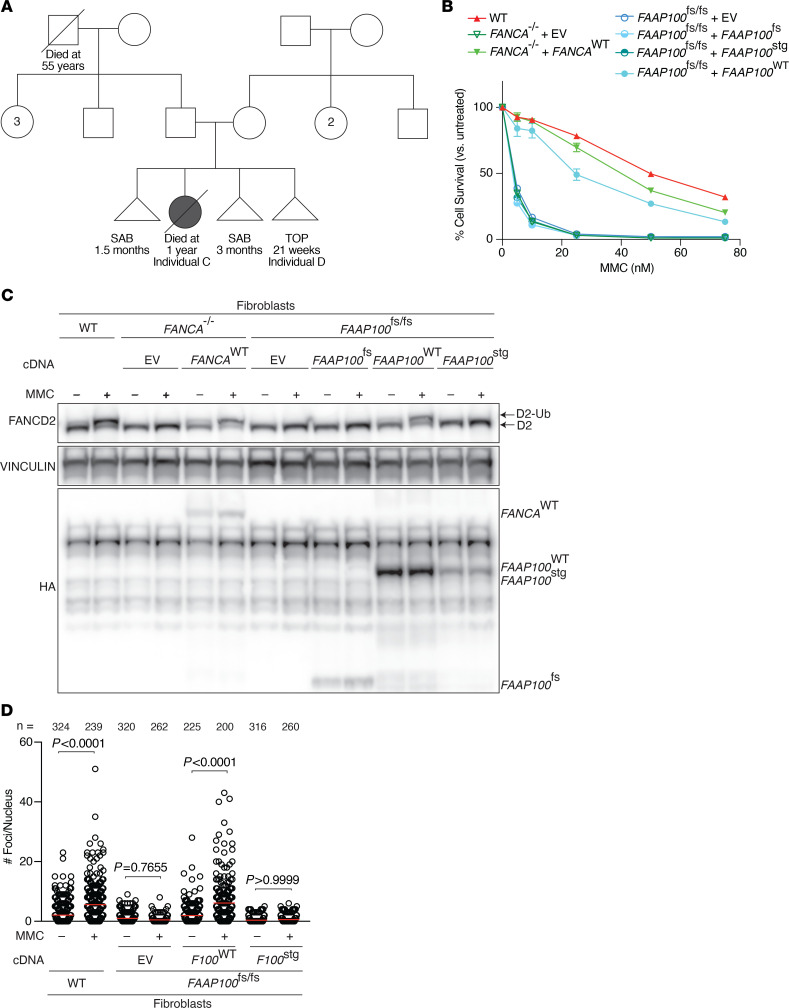
The c.2590C>T *FAAP100* variant identified in family 2 (*FAAP100*^stg/stg^) removes the last 18 amino acids from the protein. (**A**) Pedigree of family 2 with the c.2590C>T variant. TOP, termination of pregnancy. (**B**) Expression of *FAAP100*^stg^ cDNA failed to complement the MMC hypersensitivity of *FAAP100*^fs/fs^ fibroblasts. (**C**) Immunoblot showing a lack of restoration of FANCD2 monoubiquitination in cells complemented with *FAAP100*^stg^ mutant cDNA, along with confirmation of protein expression by the HA tag. (**D**) Quantification of nuclear FANCD2 foci in the indicated cells in at least 200 nuclei per cell line. Experiments were conducted at least 3 times in biological replicates with consistent results for **B** and **D**. Data from a representative experiment are shown. Data indicate the mean ± SEM. All *P* values were calculated using 1-way ANOVA.

In the couple’s fourth pregnancy (individual D), prenatal ultrasound revealed dilated lateral cerebral ventricles and thoracic subcutaneous edema. The parents elected to terminate the pregnancy. Pathology examination of the female fetus was notable for bilateral cerebral lateral ventriculomegaly, a dysplastic pancreatic tail, bilateral ectopic kidneys, and incomplete lung lobation. See Supplemental Table 1, Figure 4A, and Supplemental Text for additional clinical details on individuals C and D. Exome sequencing of individual D revealed a homozygous stop-gain variant, c.2590C>T (p.Gln864Ter), in exon 9 of *FAAP100* (NM_025161.6), leading to loss of the final 18 C-terminal amino acids. The variant is in the stretch of homozygosity, suggesting a founder effect. The variant is heterozygous in both parents.

To test the function of this stop-gain *FAAP100* variant, we expressed the mutant cDNA (*FAAAP100*^stg^) in the *FAAP100*^fs/fs^ fibroblasts derived from individual B. Presence of the truncated constructs were confirmed by immunoblotting of the HA-tag (Figure 4C, lower blot). This construct failed to complement MMC hypersensitivity (Figure 4B), FANCD2 ubiquitination (Figure 4C, upper blot), and FANCD2 nuclear foci formation (Figure 4D). These results indicate that the last 18 amino acids are necessary for the function of FAAP100 in the FA pathway.

## Discussion

In this report, we have identified what to our knowledge is a new FA subtype resulting from a deficiency of FAAP100. We identified 2 families harboring different causal variants in *FAAP100*, one of which resulted in complete loss of FAAP100 protein expression and another causing deletion of the 18 C-terminal amino acids. Despite the difference in the variants, the resulting cellular phenotypes of ICL sensitivity, FANCD2 ubiquitination, and nuclear foci formation were remarkably similar. These results are consistent with the existing structural and biochemical evidence highlighting the integral role of the FAAP100 protein in FA core complex function (13, 14, 27, 29). Furthermore, the results support the importance of the C-terminus of FAAP100, given that the loss of 18 C-terminal amino acids in the context of a protein composed of 881 amino acids is sufficient for abrogation of all in vivo activity of the protein. Even the presence of a C-terminal tag on the WT *FAAP100* cDNA expression vector resulted in failure of complementation of the *FAAP100*^fs/fs^ cells (Supplemental Figure 2C). These results emphasize that marked perturbations of FAAP100’s C-terminus, including non-native protein tags, can abrogate all protein function. Given our findings, and the findings of a pathogenic homozygous T542P variant in FAAP100 in an fetus with a phenotype consistent with FA identified by Detlev Schindler and colleagues (personal communication), the *FAAP100/FANCX* gene should be added to the existing genetic testing panels for FA and may be beneficial in genetic screens for prenatal losses.

A notable finding in individual A was the presence of bone marrow failure at day 2 of life. Neonatal bone marrow failure is a very severe manifestation of FA that has only been observed in individuals with combined mutations in *FANCA* and *ALDH2* (8, 51). Similarly, in mouse models of FA, mice with only *Fancd2* mutations exhibit a long latency to developing mild hematopoietic stem cell dysfunction, but adding an *Adh5* or Aldh2*2 mutation results in bone marrow failure and leukemia (9, 50, 53, 54). While our functional data and phenotypic finding in 2 unrelated families indicate *FAAP100* loss of function as causative of a uniquely severe phenotype, we cannot definitively exclude the possible presence of unidentified modifying genetic factors contributing to disease severity.

Additionally, the mothers of both families notably had experienced several SABs before carrying 2 infants to term. It is possible that homozygosity for the *FAAP100* variants contributed to some of these losses, but no tissue from those pregnancies was collected for genetic testing, so the genotype could not be confirmed. Given the high percentage of affected pregnancies in these families, we cannot exclude other unidentified non-FA-related genetic causes being responsible for some of the SABs.

Given the structural evidence that FAAP100 heterodimerizes with FANCB to function in the FA core complex, one would expect patients with a deficiency of either of the genes to have similar clinical presentations. However, it appears, from this report, that *FAAP100* variants are even more deleterious than the most severe *FANCB* mutations described previously (43). One explanation for this observation could be that FAAP100 is involved in other DNA repair processes outside of the FA repair pathway. This is unlikely, since previous studies using a CRISPR screen to identify genes whose loss confers sensitivity to DNA-damaging agents showed that *FAAP100* deletion results in an identical profile of sensitivity as seen with other FA pathway gene knockouts (55). Another possibility is that even without FANCB, FAAP100 can support some activity of FANCL in ubiquitination of the FANCI and FANCD2 complex during development, but without FAAP100, the pathway is completely defective. Structural work shows differential binding of FAAP100 and FANCB with other core complex components. In the context of the asymmetric core complex, there is a possibility that without FAAP100, the active side of the core complex cannot properly assemble and function (13). Very sensitive methods will be necessary to identify residual ID2 ubiquitination in cells from different patients to assess if there are meaningful differences in cells with knockouts of FANCB and FAAP100.

## Methods

### Study individuals and cell lines

#### Family 1.

Blood and skin biopsies from individual B were collected, and primary fibroblast and EBV-transformed lymphoblastoid cell lines were generated by the Johns Hopkins Genetic Resources Core Facility. Medical records for both neonates were gathered from all treating hospitals, and a complete review of these medical records was performed, including prenatal, birth, medical, and family histories along with physical examination, imaging, and laboratory tests. Additional details regarding the family history were reviewed with the parents after enrollment in the study.

#### Family 2.

No cell lines were derived from individuals in this family. Individual D in this family was found to have developmental abnormalities upon prenatal ultrasound.

See the Supplemental Text for a detailed description of both families.

### Variant identification

A heterozygous *FAAP100* variant was identified in the mother of family 1 by the “exome slice” genetic testing panel, prompting targeted reanalysis of individual A’s rGS, which uncovered a homozygous mutation in this individual. The same homozygous mutation was identified in exome sequencing of an amniocentesis sample from individual B.

The *FAAP100* variant in family 2 was identified upon exome sequencing of individual D. Subsequent Sanger sequencing confirmed that the variant was heterozygous in both parents. This variant was uploaded into GeneMatcher, which is how the authors were connected (56).

See the Supplemental Methods for detailed information on the genetic workup for both families.

### Cell culture and viral transfection/transduction

Individual B (RA3640), *FANCA*-deficient (RA3087), and BJ normal foreskin control (ATCC) fibroblasts were transformed by expression of HPV16 E6E7 and immortalized with the catalytic subunit of human telomerase (hTERT). Fibroblasts were cultured in DMEM supplemented with 15% FBS, 100 units of penicillin per milliliter, 0.1 mg/mL streptomycin, and GlutaMAX (Invitrogen, Thermo Fisher Scientific). Individual B (RA3641), *FANCA*-deficient (RA2939), and normal control (RA3536) LCLs were established from PBMCs by EBV transformation, and all LCLs were grown in RPMI with 20% FBS and further supplemented as described above. cDNAs were delivered using lentiviral transduction after packaging in HEK293 T cells according to the manufacturer’s protocol (Mirus). Fibroblasts were transduced in the presence of polybrene (4 μg/mL), selected in 2 μg puromycin per milliliter media, and maintained in 1 μg puromycin per milliliter media. RA3087 cells have a homozygous deletion of the *FANCA* locus c.793-?_4368+?del (delEx9_43) (23).

### Plasmids

WT *FAAP100* cDNA was obtained by reverse transcription of total mRNA extracted from BJ fibroblasts using the RNeasy Plus kit (Qiagen). cDNA was cloned into pDONR223 and recombined with a pCMV retroviral vector using the Gateway system (Invitrogen, Thermo Fisher Scientific), resulting in an N-terminally HA-FLAG–tagged FAAP100. Patient-derived mutant cDNAs were derived by mutagenizing WT cDNA in pDONR223 using site-directed mutagenesis according to the manufacturer’s protocol (Agilent Technologies) and subsequently recombined with a pCMV lentiviral vector using the Gateway system.

### Chromosomal breakage and cell survival analyses

Analysis of chromosomal breakage following treatment with DNA-damaging agents was performed as described in Kim et al. (22). For cell survival assays, cells were seeded overnight in triplicate and treated the next day with MMC at the indicated concentrations. Cells were grown for 3–4 days and passaged once at appropriate ratios. Once cells reached near confluence, they were counted using the Z2 Coulter counter (Beckman Coulter).

### PCR, reverse transcription, and RT-qPCR

PCR reactions were performed using Taq DNA polymerase (Qiagen), Phusion High Fidelity PCR Master Mix with GC buffer (Thermo Fisher Scientific), and PCR SuperMix High Fidelity (Invitrogen, Thermo Fisher Scientific) according to the manufacturers’ protocols, and the primers are listed in Supplemental Table 3. Total mRNA was extracted using the RNeasy Plus kit (Qiagen). Superscript III reverse transcriptase followed by Platinum SYBR Green SuperMix-UDG (Invitrogen, Thermo Fisher Scientific) was used according to the manufacturer’s protocol and normalized against *GAPDH*. The primers used for *FAAP100* cDNA amplification for RT-qPCR targeted exons 4 and 5 of the cDNA downstream of the frameshift mutation.

### Western blotting and antibodies

Whole-cell extracts were prepared by lysing cell pellets in Laemmli sample buffer (Bio-Rad) followed by sonication. Samples were boiled and separated on 4%–12% Bis-Tris or 3%–8% Tris-acetate gradient gels (Invitrogen, Thermo Fisher Scientific) by SDS-PAGE. Immunoblotting was performed using the following antibodies: FANCD2 (Novus, NB100-182, lot 8-9); HA (BioLegend, clone 16B12, lot B379455); and FAAP100 (gift from Weidong Wang, NIH, Laboratory of Genetics and Genomics, National Institute on Aging).

### IF analysis

Cells were fixed in 3.7% formaldehyde and permeabilized with 0.5% Triton in PBS, blocked in 5% (v/v) FBS in PBS, and incubated with FANCD2 antibody (Novus, NB100-182, lot 8-9) at 1:2000 dilution in blocking buffer. Cells were washed and incubated with Alexa-Fluor 488 secondary antibody (Thermo Fisher Scientific, A11008, lot 645151). Cells were washed, and coverslips were embedded with DAPI Fluoromount-G (SouthernBiotech).

### Statistics

As all experiments compared the means of 3 or more groups, 1way ANOVA followed by a multiple-comparison test was used for all statistical analyses.

### Study approval

Family 1 was enrolled in the Pediatric UDP at the NYU Grossman School of Medicine. The study was approved by the NYU Grossman School of Medicine Institutional Review Board. Work at the Rockefeller University was approved by the Institutional Review Board under protocol AAU-0112.

### Data availability

Values for all data points presented in the figures are available in the Supporting Data Values file. Sharing of the clinical genomic data was not consented to as part of this research study and thus will not be available.

### Authors contribution

EMP and GDE provided clinical data from individual B and cell lines. KT provided pathology data for study individual B. JP provided a detailed description of physical abnormalities in individual B. CSM, BDG, AW, and YK identified *FAAP100* variants and provided clinical data from individual A. TKT and GDE enrolled family 1 in the NYU UDP. SV, SH, A Shukla, and SSN diagnosed and provided details on family 2. SSN identified the *FAAP100* variants and wrote the clinical and molecular details for family 2 and acquired funding for the same. BAH and A Smogorzewska designed the experiments and assembled the figures. EMP, CSM, GDE, and BDG wrote the clinical portions of the manuscript. BAH and A Smogorzewska wrote the experimental portions of the manuscript. BAH performed all experiments. A Smogorzewska supervised experiments and acquired funding. All authors edited and approved the manuscript.

## Supplementary Material

Supplemental data

Unedited blot and gel images

Supporting data values

## Version 1 04/17/2025

In-Press Preview

## Version 2 06/02/2025

Electronic publication
